# Effects of culture media conditions on production of eggs fertilized in vitro of embryos derived from ovary of high grade Hanwoo

**DOI:** 10.1186/s40781-016-0093-5

**Published:** 2016-03-01

**Authors:** Jun Young Lee, Yun Gil Jung, Byoung Boo Seo

**Affiliations:** Department of Animal Resources, Daegu University, Gyeongbuk, 38453 Korea; Agricultural Technology Center, Gyeongbuk, 39687 Korea; ETbiotech, Jeollnam do, 57344 Korea

**Keywords:** Hanwoo ovary, *IVF*, Serum free medium, Blastocyst, Embryo transfer

## Abstract

**Background:**

This study was investigated the effects of culture media conditions on production of eggs fertilized in vitro of embryos from ovaries of high grade Korean native cow, Hanwoo.

**Methods:**

The IVMD 101 and IVF 100 were used for in vitro maturation of selected Hanwoo oocytes and In vitro embryo culture after in vitro fertilization, respectively. The IVMD 101 and IVD 101 were used for in vitro culture and completely free of serum.

**Results:**

The cleavage rates of 2-cell embryos in reference to Hanwoo oocytes were 86.7, 92.9 , and 90.1 % in the control group, IVDM101 medium and IVD101 medium, respectively which indicates that the IVDM101 medium and IVD101 medium may result favorable outcomes. The in vitro development rates of blastocysts were 12.4, 38.4 and 32.4 % in the control group, serum free IVMD101 medium and IVD101 medium, respectively. For hatched blastocysts, it was 5.3, 33.9, and 28.6 % in the control group, serum free IVMD101 medium and IVD101 medium, respectively. Hence, more favorable results were expected for the hatched blastocysts in which the IVMD101 medium and IVD101 medium were used than the control group. Average cell numbers of blastocysts were 128.3, 165.7, and 163.6 in the groups of TCM-199 + 10 % FBS medium, IVMD 101 medium, and IVD 101 medium, respectively which clearly show that the IVMD 101 and IVD 101 medium consequence significantly higher cell numbers compared to the control group (i.e., TCM-199 + 10 % FBS medium). Pregnancy rate after embryo transfer was 39.6 % when the serum free medium was used which is higher than that of the medium supplemented with serum (32.8 %). In addition, stillbirth rates were 4.9 % in the group of serum free medium whereas it was 13.6 % in the serum supplemented medium (13.6 %).

**Conclusions:**

Taken altogether, serum free media, the IVMD 101 and IVD 101 represented more favorable results in the embryo development rate of embryos, cell numbers of blastocyst, and pregnancy rate. Of note, the IVMD 101 medium showed better outcomes hence, it might be a better option for future applications for in vitro culture of bovine embryos.

## Background

Multiple studies have been carried out regarding egg transfer utilizing various animal models such as cow [1,2], sheep [3], goat [4], and pigs [5]. Ever since Brackett et al. [6] collected immature embryos from ovaries of slaughtered cows and successfully produced calve, a number of investigations was performed with regards to in vitro egg production; these studies have enabled that approximately 20 ~ 30 % of in vitro fertilized oocytes were stably developed to transferrable blastocysts. In particular, when it comes to the transfer of bovine embryos, it has been widely utilized as a measure to secure excellent genetic traits since it harvests multiple eggs from female cows with great genetic traits and produces offspring after transfer into other cows [7].

It was reported successful transfer of in vivo fertilized embryos of bovine [8–10]. To date, most techniques of in vitro embryos production were relied on cows already slaughtered hence they are limited in a way that genetic traits of maternal bovine were not known (Rosenkrans et al., [11–13]; Hasler et al., [14]; Blondin et al., [15,16]). In recent, even though techniques for embryos transfer have been commercialized, these are mostly limited to transfer of in vivo fertilized eggs in Korea thus further improvements are warranted. In vitro embryos are important genetic resources of Hanwoo, yet have significantly contributed to production thereof. Therefore, improvement of livestock might be meaningfully enhanced if in vitro embryos are individually separated depending on their genetic abilities and then, oocytes derived from an animal with great quality and amount of meats are selectively cultured. Regardless of in vitro and/or in vivo embryos, selection of maternal bovine is critical in regards to improvement of livestock; industrial values of in vitro eggs might be widely recognized in which all information of pedigree, ability, and carcass information of livestock are available that may indicate meat productivity of donor cows. On the other hand, in vitro fertilization also represents limitations: relatively low conception rate, miscarriage, and stillbirth. In Japan, these issues have been improved and resolved by the government through active efforts on development of in vitro culture techniques; when compared the meat quality of offspring, the production rate of high quality meat was higher than artificial fertilization, making farmers to profit from it and to prefer this method over the artificial fertilization.

In the present study, we aimed to pursue massive production of in vitro fertilized embryos of Hanwoo that possess economic traits of high quality of meat; in order to achieve the objective, embryos were harvested from ovaries of slaughtered Hanwoo with excellent quality of meats (1, 1+ and 1++ grade) and then cultured in different media to investigate overall impacts on the production of in vitro fertilized eggs such as in vitro embryo development rate, cell numbers of blastocysts, and pregnancy rate.

## Methods

### Harvesting ovaries of Hanwoo with high quality of meats

Hanwoo cows were slaughtered by the Samwa industry (Ulsan, South Korea) and then, ovaries were collected from each animal; blood and other foreign matters were removed and then ovaries were stored in thermos bottles, coded with respective serial number for each animal. Collected ovaries were immersed in normal saline solution and then transferred to the lab within 3 h. Ovaries were further screened for the further analyses only if the meat grade is 1 or higher (e.g., 1+ or 1++) which was determined by comparing cattle scorecards, issued by the Meat Quality Judge, and the serial numbers for respective animals. Selected ovaries were then further classified into either 1) Basic registration, 2) Pedigree registration or 3) Non-classified by tracking bar-code of each animal through Hanwoo entity database of the Association of Breed Improvement.

### Culture media

For embryo harvest, the tissue culture medium-199 (TCM-199) supplemented with 0.3 % bovine serum albumin (BSA) was utilized. The IVMD 101 (Functional Peptides Research Institute, Japan) and IVF 100 (Functional Peptides Research Institute) were used for in vitro maturation of oocytes and in vitro fertilization, respectively. The IVMD 101 and IVD 101 were used for in vitro culture and completely free of serum (Functional Peptides Research Institute, Japan).

In order to clean sperms and in vitro fertilization, the IVD 100 medium was supplemented with BO medium which was slightly modified by adding 25 mM sodium pyruvate, 0.5 mM cysteine, 5 mg/mL BSA, 5 mM caffeine (Wako Pure Chemical Industries, Ltd, Japan), and 7.5 mg/mL heparin. The complete compositions of the IVD 101 and IVDM 101 were shown in the Table 1 [17].

**Table 1:** Composition of IVD 101 medium and IVMD 101 medium^a^

Components	IVD 101	IVMD 101
D-glucose(mM)	2.22	5.56
Sodium pyruvate(mM)	0.27	0.91
Sodium lactate(mM)	2.48	
L-cysteine(mM)	0.05	
GSH(μm)	200	
Taurine(mM)	5	5
Selenium(mM)	5	5
Insulin(ug/Μℓ)		5
TGF-a(mg/Μℓ)		10
Apo-transferrin(ug/Μℓ)	10	10
bFGF(mg/Μℓ)	10	
TGF-B1(mg/Μℓ)	1	
TIMP-1(ug/Μℓ)	0.5	
Aprotinin(ug/Μℓ)	0.5	
BSA(mg/Μℓ)	1	1
HEPES(mM)	5	5
Gentamycin sulfate(ug/Μℓ)	10	10

^a^Basal medium of IVD 101 and IVMD 101

All media were prepared 2 weeks prior to the experiments and then filtered using the 0.22 μm membrane filter (Gelman Sciene, USA); the filtered media were stored at 4 °C and used after 4 ~ 5 h equilibrium at 5 °C in a CO_2_ incubator.

### Collection of embryos

Ovaries harvested from Hanwoo slaughtered in a slaughter house were washed 2 ~ 3 times using normal saline and then remaining blood and foreign bodies on the surface were further cleaned. Embryos were harvested from 2 ~ 6 mm oocyte follicle using a 10 mL syringe with 18 gauge needle. The oocytes were then washed with the TCM-199 solution 2 ~ 3 times and the supernatant solution was removed. Utilizing a Pasteur pipette, embryos with securely attached with cumulus cells were selected under a stereoscopic microscope and then sub-cultured in a 35 m culture dish per each animal.

### In vitro maturation of oocytes

Harvested oocytes per each animal were washed 1 ~ 2 times using the IVMD 101 medium and then aliquoted into a 5-well dish (800 μL per each well; 20 ~ 40 oocytes per animal per well) and then in vitro maturation was performed in an incubator maintaining 39 °C and 5 % CO_2_ over 22 ~ 24 h. Oocytes with well dispersed cumulus cells were chosen for further in vitro fertilization.

### Sperm preparation and in vitro fertilization

Sperms were obtained from the National Agricultural Cooperative Federation Livestock Improvement Office (Serial number: KPN 413, 538 and 676) and used for in vitro fertilization. This frozen semen was sit at the room temperature for 10 s and then thawed in 39 °C water for 20 s. Using a sterilized scissors, both sides of straw were cut and semen solution was transferred into a 15 mL centrifuge tube (Falcon, 2097) containing 4 mL IVF 100 solution; the solution was centrifuged at 700 rpm for 5 min and resulting supernatant was discarded except approximately 200 μL solution, remained on the bottom. Again 4 mL of IVF 100 solution was added into the solution and centrifuged at 700 rpm for 5 min. After discard the resulting supernatant, the final concentration of semen solution was reconstituted to 1 × 10^7^/mL. The in vitro fertilization was carried out using embryos, matured for 22 h in 100 μL drop of IVF coated with mineral oil; after washing prepared embryos 1 ~ 2 times, the semen was subjected and then in vitro fertilization was induced on a 60 mm petri dish over 6 h in an incubator maintaining 39 °C and 5 % CO_2_.

### In vitro embryo culture

Upon completion of in vitro fertilization over 6 h, embryos were transferred using a 200 μL micropipette and cumulus cells were removed except for three layers. Under the stereoscopic microscope, shape of embryos was monitored and satisfactory ones were further harvested per each animal. Collected fertilized embryos were washed 2 ~ 3 times with 100 μL drop of IVMD 101, coated with mineral oil, on a 6 mm petri dish. One hundred microliter drop of IVMD 101, containing 20 ~ 40 embryos, per each animal was cultured in vitro for 24 h on a 60 mm dish (Falcon, 3002). Attached cumulus cell layers (approximately three layers) were completely removed after 30 h of in vitro incubation and then fertilized embryos were aliquoted into a 6-well dish (200 μL per each well), sprayed with mineral oil, and then cultured in the IVD 101 medium in a low oxygen incubator (5 % oxygen). Fertilized embryos were subjected to medium change on 5^th^ day of fertilization (50 % medium change; 100 μL of fresh IVD 101 solution per each well) and further maintained until 7 ~ 8^th^ day to confirm development of blastocysts.

### Statistical analysis

All experimental results were expressed as percentage and statistical analyses were performed utilizing the Statistical Analysis System (SAS Institute, 9.2 Version). One way analysis of variance was performed followed by the Duncan’s multiple range tests to examine differences between groups. A *p* value less than 0.05 was considered statistically significant.

## Results

### Comparison of in vitro development rates between medium containing serum versus serum free media

In the present study, the TCM 199 medium containing 10 % FBS was utilized as a control group. The serum free media include the IVDM 101 and IVD 101 that are supplemented with energy source and cell growth factors. In order to compare in vitro development rates between medium containing serum (i.e., control group) versus media free of serum supplementation (i.e., IVDM101 and IVD 101), numbers of embryos developed to 2-cell, blastocyst, and hatched blastocyst were monitored.

As shown in the Table 2, the 2-cell cleavage rates for oocytes, derived from the Hanwoo, were 86.7 %, 92.9 %, 90.1 % in the control group, IVDM101 medium group, and IVD101 medium group, respectively. The IVDM 101 and IVD101 media represented significantly higher percentage rates compared to the control group.

**Table 2:** Comparison of Hanwoo embryos developed in serum-free culture medium and culture medium with serum after in vitro fertilization

In vitro culture medium	No. of oocytes cultured	No. (%*) of embryos developed to		
		2-cell	Blastocyst	Hatched blsatocyst
TCM-199				
+10 % FBS	226	178(86.7)	28(12.4)^a^	12(5.3)^a^
IVMD 101	224	208(92.9)	86(38.4)^b^	76(33.9)^b^
IVD 101	284	256(90.1)	92(32.4)^b^	82(28.6) ^b^

*Percentage of the number of embryos cultured

^a,b^Values with different superscripts in the same column were significantly different (*p* < 0.05)

In addition, when it comes to the development rates of blastocyst, it was 12.4 %, 38.4 %, and 32.4 % for the control group, IVMD 101 medium group, and IVD 101 medium group, respectively which is also in agreement with results of 2-cell cleavage rates; both serum free media showed more favorable results compared to the serum supplemented medium. No significant difference in the development rate of blastocyst was noted between the IVMD 101 and IVD 101 groups. In the development rate of hatched blastocyst, the control group, IVMD 101 medium group, and IVD 101 medium group had 5.3 %, 33.9 %, and 28.6 %, respectively. Likewise, the serum free media groups (i.e., IVMD 101 and IVD 101 groups) had higher development rates of hatched blastocyst. Overall, embryo development rats were higher in the serum free media, IVMD 101 and IVD 101 media, compared to those of control group. Further, the IVMD101 medium group showed significantly higher development rates than the IVD 101 group as well.

### Comparison of cell numbers of Hanwoo blastocysts between medium containing serum versus serum free media

Cell numbers of Hanwoo blastocyst, developed in either serum free medium (i.e., control group) or serum supplemented media (i.e., IVMD 101 and IVD 101 groups) were compared after 7 ~ 9 days of in vitro incubation. As indicated in the Table 3, the average cell numbers of Hanwoo blastocysts were 128.3, 165.7, and 163.6 in the control group, IVMD 101 medium group, and IVD 101 medium group, respectively. This result clearly shows that cell numbers of Hanwoo blastocysts were significantly higher in the tested groups (IVMD 101 medium and IVD 101 medium) compared to the control group. No statistical difference was shown between the IVMD 101 and IVD 101 groups. Further, we also noted that cell division speed of fertilized embryos in serum free media was much faster than the control group and similar cell numbers were shown on the 7^th^ day of fertilization compared to the in vivo fertilization (data not shown).

**Table 3:** The cell number of Hanwoo blastocysts developed in serum-free culture medium and culture medium with serum after in vitro fertilization

In vitro culture medium	No. of Blastocyst	Total cell no.
TCM 199		
+10 % FBS	16	128.3^a^
IVMD101	18	165.7^b^
IVD101	19	163.6^b^

^a,b^Values with different superscripts in the same column were significantly different

(*p* < 0.05)

### Comparison of pregnancy rate between medium containing serum versus serum free media

The pregnancy rate, birth rate, abortion rate, and stillbirth rate were monitored after embryo transfer of Hanwoo blastocysts developed in either serum free medium or culture media free of serum. In the control group, the pregnancy rate, birth rate, abortion rate, and stillbirth rate were 32.8 %, 86.4 %, 13.6 %, and 13.6 %, respectively. In contrast, in the group of serum free media, they were 39.6 %, 85.2 %, 14.8 %, and 4.9 %, respectively (Table 4). As shown, the pregnancy rate of Hanwoo blastocysts developed in serum free media was significantly higher than the control group (39.6 % vs. 32.8 %). In addition, their stillbirth rate was lower than the control group as well (4.9 % vs. 13.6 %). Therefore, our results show that serum free media represent better outcomes in pregnancy rate and stillbirth rate compared to those of serum containing culture medium, TCM 199 + 10 % FBS.

**Table 4:** Comparison of pregnancy rate, Birth rate, abortion rate, and stillbirth rate after embryo transfer of Hanwoo blastocysts developed in serum-free culture medium and culture medium with serum

In vitro culture medium	No. of recipients	Pregnancy rate (%)	Birth rate rate (%)	Abortion rate (%)	Stillbirth rate (%)
TCM 199					
+10 % FBS	67	22/67 (32.8)	19/22 (86.4)	3/22 (13.6)	3/22 (13.6)
IVMD 101					
/IVD 101	154	61/154 (39.6)	52/61 (85.2)	9/61 (14.8)	3/61 (4.9)

## Discussion

In the present study, the control group was the TCM 199 medium supplemented with 10 % FBS, whilst the serum free media were IVDM 101 and IVD 101 containing energy source and cell growth factors. Once Hanwoo oocytes were fertilized in vitro, their development rates were compared by monitoring numbers of embryos developed to 2 cell, blastocyst and hatched blastocyst. As results, the 2-cell cleavage rates were 86.7, 92.9, and 90.1 % in the control group, IVDM 101 medium group, and IVD 101 medium group, respectively. According to the study of [18], the 2-cell (or higher) cleavage rates were 66.4 % and 62.4 % in Hanwoo and dairy cattle, respectively. In another studies, Bondioli et al. [19] and Kim et al. [20] also reported 40 % and 50 % cleavage rates. When it comes to the development rate of blastocysts, that were harvested from Hanwoo, fertilized and cultured in vitro, Izadyare et al. [21] reported 28.2 %. In another study, [22] also reported 27.0 % and 23.4 % of development rates in Hanwoo and dairy cattle, respectively. On the other hand, survival rate of fertilized embryos after vitrification freezing in response to culture conditions was compared; embryos from slaughtered Hanwoo ovaries were fertilized in vitro and cultured at 38.5 °C and 5 % CO_2_ conditions. The cleavage rates of the eggs were 73.2, 69.3, and 72.8 % in the CRlaa culture medium, IVDM medium, and IVD medium, respectively. However no statistical significance was noted between groups [23]. Similar to aforementioned evidences in literature, we were able to demonstrate that the 2-cell cleavage rates in the IVDM 101 and IVD 101 groups were higher than those of the control group. When it comes to the development rates of blastocysts of Hanwoo, the tested groups (IVMD 101 medium and IVD 101 medium) resulted 38.4 % and 32.4 %. In recent, Cho et al. [23] demonstrated that there was no difference in development rates of blastocysts between tested media (i.e., IVDM 101 medium vs. IVD 101 medium; 30.8 % vs. 33.3 %) which is somewhat similar results herein. Lastly, the development rates of hatched blastocysts were 5.3, 33.9, and 28.6 % in the control group, IVMD 101 medium group, and IVD medium group, respectively. The serum free media groups showed significantly higher development rates of hatched blastocyst than the control group. Further, we noticed that cell division speed of fertilized embryos was much faster in the serum free media compared to the control group. Cell numbers shown on 7^th^ day of fertilization were similar to those of in vivo fertilization as well. When compared to the average Hanwoo blastocysts, our results indicate there is no noticeable difference shown. Hanwoo blastocysts developed in serum free media (IVMD 101 and IVD 101 media) represented higher pregnancy rate compared to the control group (39.6 % vs. 32.8 %). In contrast, the stillbirth rate in the serum free group was much lower than the control group (4.9 % vs. 13.6 %). It has been widely accepted that development rates of blastocysts could vary even though similar in vitro fertilization conditions are subjected; however the pregnancy rates are expected to be similar despite of different culture systems (52.8 % and 54.5 in the TCM culture and B2 culture system, respectively) [24]. Compared to those previous results, our pregnancy rates are somewhat low and these might be partially contributed by individual differences and their technical skills.

Taken altogether, serum free media, the IVMD 101 and IVD 101 showed more favorable results in the embryo development rates cell numbers of blastocyst and pregnancy rate. In particular, the IVMD 101 medium showed better outcomes hence might be more suitable for future applications.

## Conclusion

Taken altogether, serum free media, the IVMD 101 and IVD 101 represented more favorable results in the embryo development rate of embryos, cell numbers of blastocyst, and pregnancy rate. Of note, the IVMD 101 medium showed better outcomes hence, it might be a better option for future applications for in vitro culture of bovine embryos.
